# Assessment of a biometric shirt for sleep body position identification in epilepsy

**DOI:** 10.3389/fneur.2025.1662988

**Published:** 2025-11-07

**Authors:** Emmanuelle Nguyen, Manon Robert, Tian Yue Ding, Oumayma Gharbi, Amirhossein Jahani, Jérôme St-Jean, Claudia Rodriguez, Isabel Sarzo Wabi, Daniel Alejandro Galindo Lazo, Dang Khoa Nguyen, Elie Bou Assi

**Affiliations:** 1 Research Centre of the University of Montreal Health Centre, Montreal, QC, Canada; 2 Faculty of Medicine and Health Sciences, McGill University, Montreal, QC, Canada; 3 Department of Neuroscience, University of Montreal, Montreal, QC, Canada; 4 Institute of Biomedical Engineering, University of Montreal, Montreal, QC, Canada; 5 Neurology Division, University of Montreal Health Centre, Montreal, QC, Canada

**Keywords:** epilepsy, SUDEP, wearable devices, sleep position, prone sleeping, body position tracking, Hexoskin

## Abstract

**Background:**

Patients with uncontrolled epilepsy are at increased risk of sudden unexpected death in epilepsy (SUDEP). Evidence suggests that sleeping prone or being in a prone position after a seizure may increase the risk of SUDEP. A few wearable devices have the potential to track sleeping habits. These devices could eventually be used to screen patients with epilepsy with a tendency to sleep in a prone position, allowing interventions such as sleep training to influence an ideal sleep position. Additionally, they could continuously monitor body positioning, allowing for responsive alarms and/or interventions when necessary. In this study, we prospectively assessed the accuracy of the Hexoskin biometric shirt algorithm in identifying sleep body positions.

**Methods:**

Patients were recruited at the University of Montreal Health Center (CHUM) epilepsy monitoring unit and were asked to wear the Hexoskin biometric shirt. A built-in algorithm identified prone, supine, right, left, or sitting/standing body positions using an accelerometer. Sleeping positions predicted by the algorithm were compared to “true” values collected via blind simultaneous video analysis.

**Results:**

Across 10 patients and 347 h of sleep analyzed, 65% of prone, 75% of supine, 94% of right lateral decubitus, 81% of left lateral decubitus, and 65% of sitting/standing positions were correctly classified by the Hexoskin algorithm. Balanced accuracy was 0.76 and weighted F1-score was 0.85.

**Conclusion:**

Our results show promise in the use of the Hexoskin shirt for detecting sleep positions. Optimizing performance in identifying prone sleep could enhance its clinical utility for monitoring patients with epilepsy.

## Introduction

Sudden unexpected death in epilepsy (SUDEP) is a frequent cause of non-accidental and non-suicidal death amongst patients with uncontrolled epilepsy and constitutes a major concern for people with epilepsy (PWE) (1). Growing literature suggests that sleeping prone and being in the prone position in the post-ictal phase might be a risk factor for SUDEP and that strategies should be adopted to influence ideal positioning of PWE during the night (2–4). Prone position was in fact linked to possible fatal airway obstruction and hypoventilation, leading to SUDEP (5, 6). A meta-analysis conducted in 2015, which compiled 253 cases of SUDEP across 25 publications, found that 73.3% of the documented SUDEP victims died in a prone position (7). Therefore, sleep positions are crucial to preventing unwanted events. A few wearable devices have shown promising performances for tracking sleeping habits. Amongst these figure pressure sensors placed underneath mattresses (8), as well as triaxial accelerometers incorporated in patch-type devices applied to the chest (9), in smartphones strapped against the sternum (10, 11), in wireless audio-motion sensors placed on the neck (12), in belt-type systems (9), and in neck-based printed circuit boards (PCB) attached with double-sided adhesive (13). These devices could eventually be used to screen patients with a tendency to prone sleeping or to continuously monitor body positioning, in hopes of allowing interventions such as ideal sleep position training or responsive alarms. The Hexoskin Smart Shirt is a wearable biometric garment equipped with three electrocardiography (ECG) electrodes to record heart rate, two respiratory inductance bands across the thorax and abdomen to measure breathing rate, and a built-in 3-axis accelerometer to track motor activity. Our team recently showed that the Hexoskin shirt could detect tonic-clonic seizures in PWE by monitoring heart and movement changes (14). In this study, we aim to assess the performance of the Hexoskin smart wear to track sleep body positioning of PWE.

## Materials and methods

Patients were recruited at the epilepsy monitoring unit (EMU) of the University of Montreal Health Centre (CHUM) from June 19th, 2023, to July 4th, 2024. Eligible participants were patients over 18 years old that were admitted to the EMU for monitoring for at least 48 hours. We excluded those undergoing intracranial EEG recordings and those presenting with physical difficulties in wearing the shirt. The patients included in the study were asked to wear the Hexoskin biometric shirt continuously throughout their participation. The Hexoskin has a built-in algorithm to predict one of the following body positions using the raw 3-axis accelerometer data: prone, supine, right, left, and sitting/standing.

**Figure 1:**
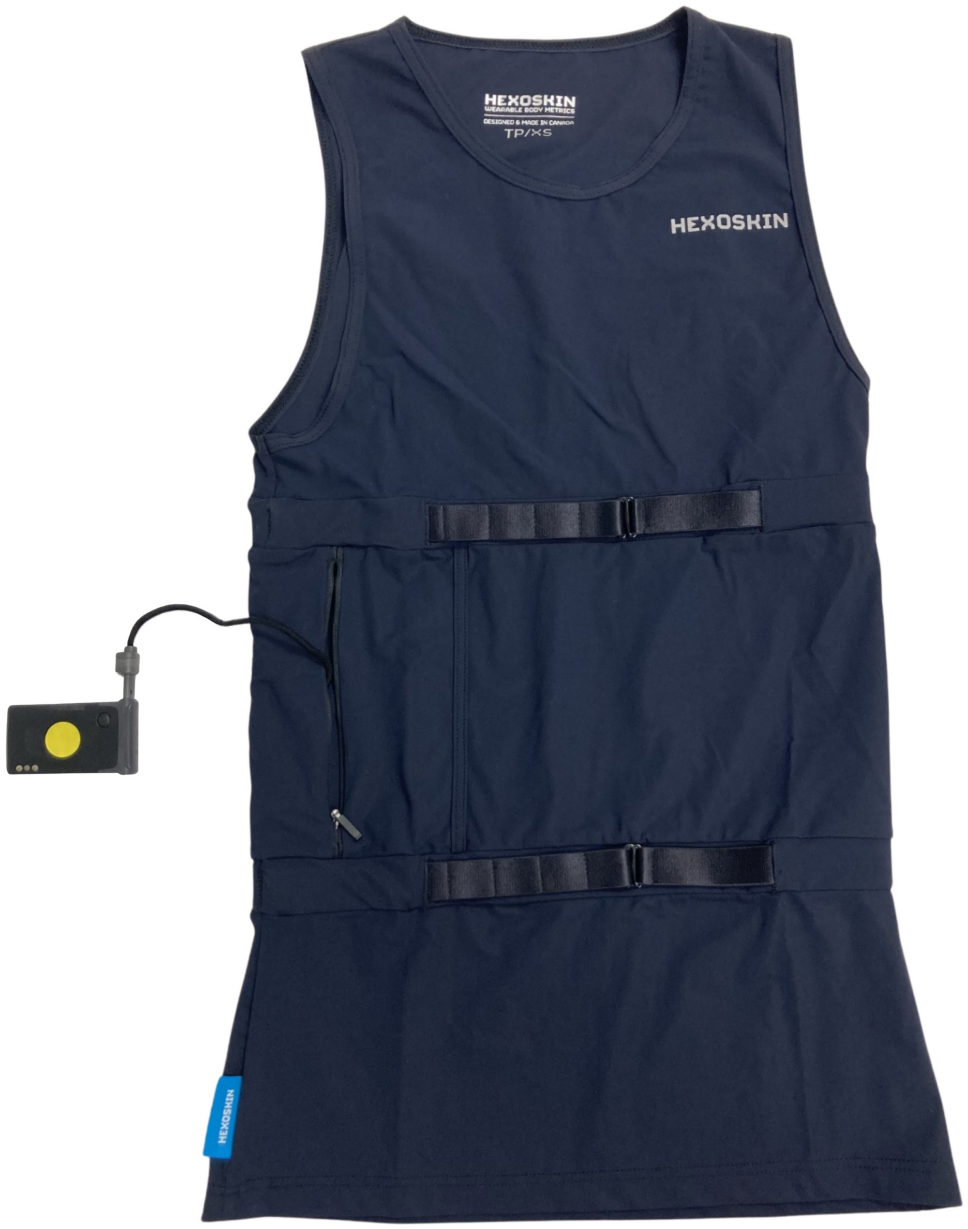
Device orientation and position inside the Hexoskin shirt.

Acquisition devices were fixed in a determined orientation inside the Hexoskin shirt pocket. We added Velcro tape strips to ensure immobility during position changes. The device was positioned following orientation recommendations indicated by the manufacturer: the device positioned on its side, with LED lights facing away from the body and device connector cable pointing upwards (Figure 1). As the device was replaced regularly to transfer the data, instructions were given to technicians and other staff to place the device in the same predetermined position. Each was asked to show the device placement on camera and report the position during each visit to ensure consistency. Data from patients were eligible for analysis if the shirt was worn correctly and the accelerometer device was correctly positioned upon inspection by the data acquisition team. Hexoskin data collected each day, excluding on nights of sleep deprivation, were downloaded as comma-separated value (CSV) files including timestamps for sleep position changes. Sleep positions were interpreted using a chart provided by Hexoskin, from sleep onset (intention to sleep) to offset (intention to wake), each based on video recordings (Table 1).

**Table 1:** Chart for interpreting sleep position based on device orientation.

**Interpretation of sleep positions**	
**Hexoskin data**	**Corresponding position**
1	Prone
2	Supine
3	Right
4	Left
5	Standing or sitting

To analyze the performance of the Hexoskin sleep position detection algorithm, the previously extracted timestamps of position changes were used to assign a position of the body to each second of the analyzed sleep. The true body positions of the patients were annotated by the first author (EN) according to continuous video-electroencephalography (VEEG) recordings at the EMU, blindly to the wearable shirt data. An independent team member (MR) further reviewed positions deemed more difficult to categorize (15). As international consensus has not yet been established to categorize sleep positions, they were defined as follows, according to a study by Wang et al. (16) and further cited by Oguz Akarsu et al. (17):

a) Prone: laying on the abdomen, upper body lifted less than 60° from the horizontal plane;
b) Supine: laying on the back, upper body lifted less than 60° from the horizontal plane;
c) Lateral: laying on the side, either specified left or right, upper body lifted less than 60° from the horizontal plane;
d) Sitting: upper body lifted more than 60° from the horizontal plane;
e) Standing.

True VEEG-based annotations and Hexoskin classifications were then synchronized based on the universal clock.

**Figure 2:**
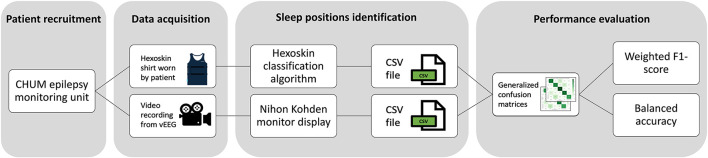
Flowchart of data acquisition, analysis, and performance assessment.

We focused on the orientation of the upper body, or the trunk more precisely, to characterize sleep positions. Indeed, the present study revolves around the possible risks of SUDEP and OSA associated with sleeping in certain positions, both linked to airway obstruction. Sleep positions based on VEEG recordings were considered true values (prone, supine, right, left, sitting/standing) and sleep positions determined by Hexoskin were considered predicted values. Multiclass confusion matrices were generated for each night where sleep was analyzed for each patient. A final normalized confusion matrix summarized data obtained for all patients and nights. Figure 2 shows the flowchart of data acquisition, analysis, and performance assessment. To evaluate classification performance, we calculated the balanced accuracy and the weighted F1-score. Balanced accuracy represents the average of the recall obtained on each class, thereby accounting for potential class imbalance. The F1-score combines both precision and recall into a single metric by computing their harmonic mean, reflecting both false positives and false negatives.

## Results

**Figure 3:**
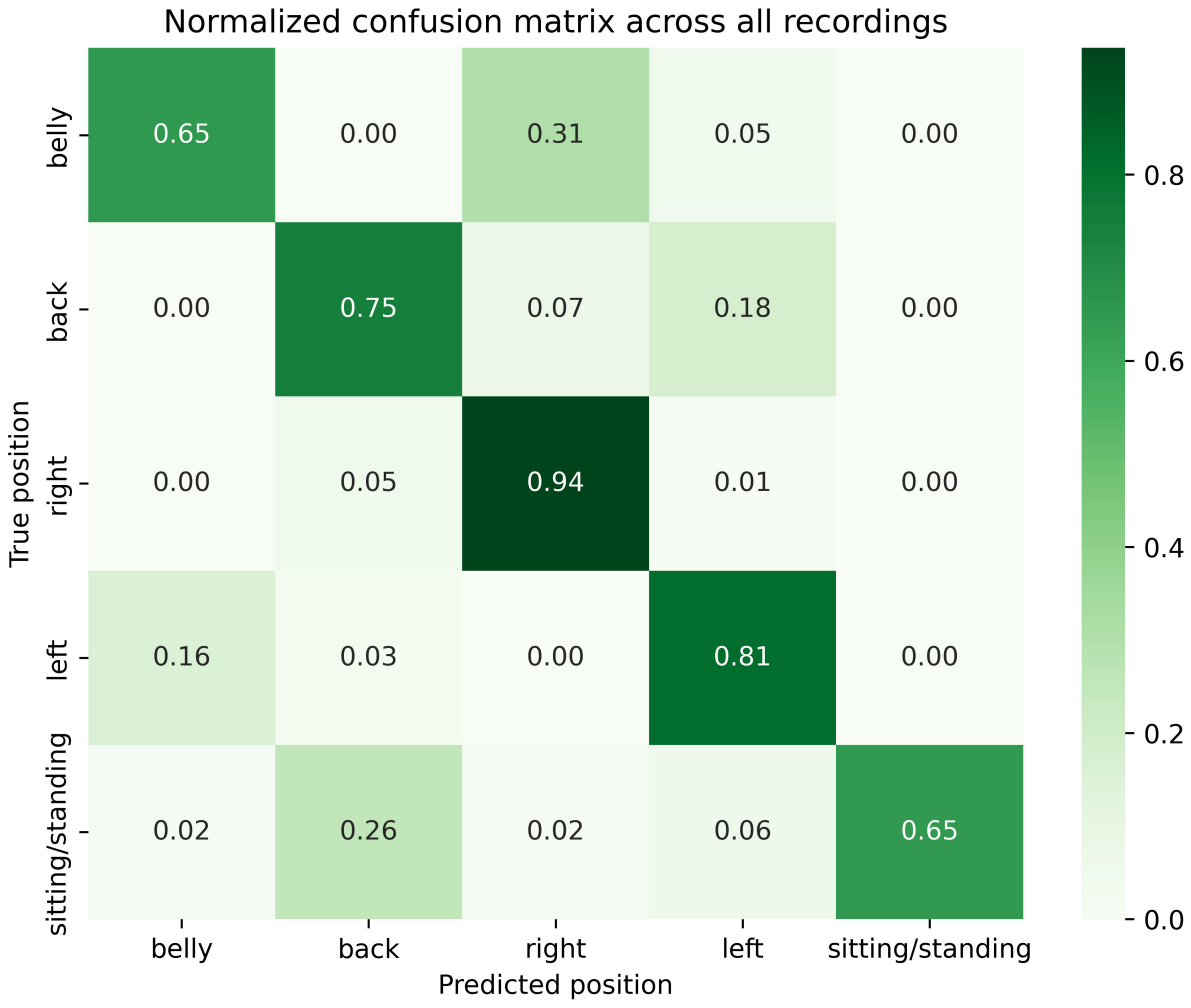
Normalized confusion matrix across all patients and nights.

In this study, we performed a prospective validation of body sleep position detected by a biometric shirt, against VEEG-based annotations in an EMU setting. In total, 10 patients were recruited in this study (Table 2). Across 65 nights analyzed via VEEG, 25 were excluded due to sleep deprivation (ordered by the treating physician to induce seizures) and technical issues (synchronization issues with the VEEG display system, missing segments of video recordings, and instances where the camera was not directed at the patient). The remaining 40 nights were analyzed for a total duration of 347 h (1,249,633 s), where most of the time was spent in the left (34%) and right (33%) lateral decubitus positions, followed by the supine position (31%), then the prone position (1%). The frequency of those different sleep positions aligned with previous data, showing a similar distribution of time spent in each sleep position (18). As shown in Table 3, the distribution of sleep positions also varied considerably between individuals: while some participants spent the vast majority in a single position (e.g. patient 1 slept 77% supine; patient 9 spent 70% in right lateral decubitus), others alternated more evenly between positions, reflecting heterogeneous individual sleep patterns. Table 3 shows the distribution of sleeping positions across individual patients, reported as percentages. Figure 3 shows the normalized confusion matrix summarizing performances of the Hexoskin detection algorithm across all patients and nights. Overall, 65% of prone, 75% of supine, 94% of right lateral decubitus, 81% of left lateral decubitus, and 65% of sitting and standing positions were correctly detected by the Hexoskin algorithm. False negative positions were detected by Hexoskin as right (31%) and left (5%) for prone position; right (7%) and left (18%) for supine position; supine (5%) and left (1%) for right position; prone (16%) and supine (3%) for left position; and prone (2%), supine (26%), right (2%) and left (6%) for sitting and standing positions. Balanced accuracy was evaluated at 0.76 and weighted F1-score at 0.85.

**Table 2:** Patient characteristics (*n* = 10).

**Patient number**	**Sex**	**Age (years)**	**BMI (kg/m^2^)**	**Diagnosis**	**Medications**
Patient 1	Male	29	32.28	Functional dissociative seizures	None
Patient 2	Male	44	24.77	Right temporal epilepsy	lacosamide, carbamazepine, topiramate, lorazepam
Patient 3	Female	28	23.81	Functional dissociative seizures	lamotrigine, amitriptyline, lisdexamfetamine, sumatriptan, isotretinoin
Patient 4	Female	18	23.89	Right temporal epilepsy	lacosamide, levetiracetam
Patient 5	Male	27	26.39	Right temporal epilepsy	levetiracetam, carbamazepine
Patient 6	Female	24	21.60	Idiopathic generalized epilepsy/frontal epilepsy	levetiracetam, carbamazepine
Patient 7	Female	30	21.63	Right focal epilepsy	carbamazepine
Patient 8	Female	32	21.26	Left temporal epilepsy	lamotrigine
Patient 9	Male	53	22.60	Left focal epilepsy	lamotrigine
Patient 10	Male	19	28.28	Left temporal epilepsy	lamotrigine, cenobamate, lacosamide

Median age was 28.5 years (range 18 – 53), 6 participants out of 10 were female, and median BMI was 23.85 kg/m^2^ (range 21.60 – 32.28).

**Table 3:** Distribution of sleeping positions across individual participants.

**Patient number**	**Percentage of time spent in each sleeping position per patient (%)**				
	**Prone**	**Supine**	**Right**	**Left**	**Standing/sitting**
Patient 1	0.02	24.16	25.17	50.55	0.10
Patient 2	0.00	44.45	27.47	24.72	3.37
Patient 3	0.00	76.89	23.09	0.00	0.02
Patient 4	0.00	37.28	26.78	35.78	0.16
Patient 5	0.00	50.10	10.43	39.44	0.03
Patient 6	0.00	23.05	66.47	10.44	0.03
Patient 7	3.82	22.28	20.51	53.23	0.16
Patient 8	0.00	48.97	29.59	21.39	0.06
Patient 9	0.00	3.98	70.06	25.85	0.11
Patient 10	3.83	22.15	33.99	39.91	0.12
Overall	1.08	31.09	33.03	34.44	0.35

**Figure 4:**
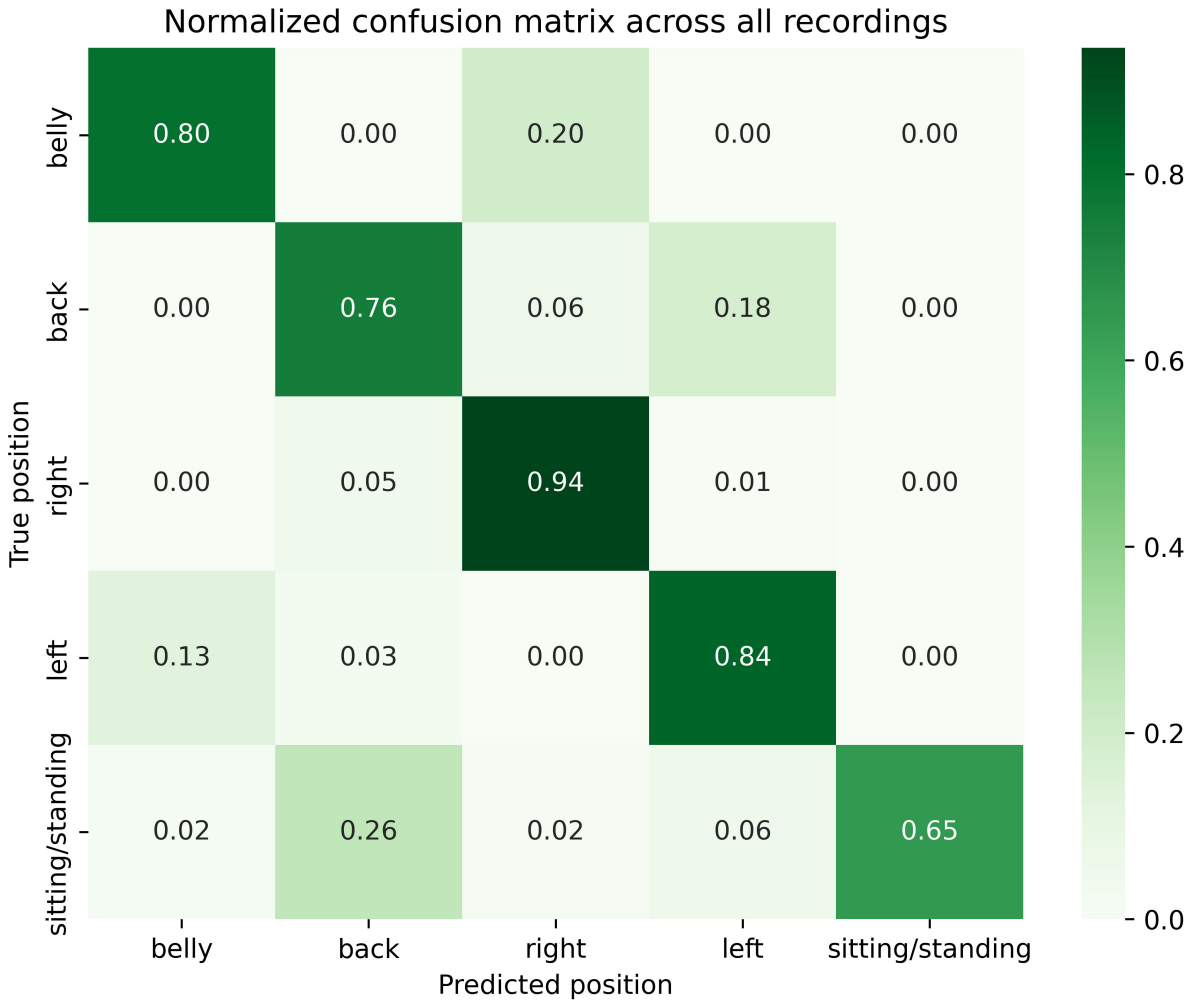
Sub analysis normalized confusion matrix across all patients and nights.

Because many positions could have been interpreted as intermediate (e.g., halfway between supine or prone and a left or right lateral decubitus position), a sub-analysis was conducted after excluding these segments, resulting in 336 h (1,210,839 s) of data available for analysis. Figure 4 shows the normalized confusion matrix summarizing performances across all patients and nights, minus times of intermediate positions. Overall, results appeared slightly more promising: 80% of prone, 76% of supine and 84% of left lateral decubitus sleeping positions were correctly detected by the Hexoskin algorithm. Specifically, only 20% of prone positions were detected as right lateral decubitus (compared to 31%), no prone positions were detected as left lateral decubitus (compared to 5%), only 6% of supine positions were detected as right (compared to 7%); and only 13% of left positions were detected as prone (compared to 16%). Balanced accuracy was reevaluated at 0.80 and weighted F1-score at 0.86.

In addition, all participants completed a questionnaire inquiring about potential usability concerns. None of the patients reported any issues with wearing the Hexoskin shirt or experiencing side effects.

## Discussion

SUDEP is the most serious adverse event that may occur in epilepsy, with drug-resistant patients experiencing tonic-clonic seizures being at the highest risk (7). For this reason, researchers have been actively developing connected devices, such as the Empatica smartwatch and the Emfit bed sensor, to detect seizures at home and enable timely intervention. Our group has recently demonstrated that the Hexoskin biometric shirt could also be a promising alternative for unobtrusive, wearable seizure monitoring. Emerging data suggest that SUDEP may be linked to prone positioning during sleep or in the post-ictal phase; recommendations include sleep training to avoid sleeping in the prone position while at risk of SUDEP (2–4). Similarly, recent developments in the field of positional obstructive sleep apnea (pOSA), another condition linked to sleep position, have introduced vibrotactile devices (i.e., devices affixed to the neck or chest that provide vibrotactile feedback when a non-optimal sleeping position is detected) in the context of positional therapy, which have been shown to improve alleviation (19, 20). Whilst posture-feedback tools have shown efficacy in other sleep disorders, there are, to our knowledge, no controlled studies to date demonstrating that such systems would decrease peri-ictal or post-ictal prone positioning in epilepsy (19, 20). Our findings provide a framework for future interventional studies integrating real-life posture detection, sleep training, and timely repositioning aimed at minimizing peri-ictal and post-ictal prone time. The goal would be eventually to use wearable devices synchronized to a mobile application for practical real-life data visualization to monitor sleeping positions in patients' home environments and to introduce interventions against prone positioning in those at risk. While baby monitors or video systems enable visual supervision, they depend on active caregiver monitoring and do not provide quantitative or automated position analysis. Wearable devices such as the Hexoskin shirt may therefore complement these systems by offering continuous and potentially alert-based monitoring. This could also be applied in other circumstances, such as positional therapy in pOSA. In this optic, this study aimed to evaluate the accuracy of the Hexoskin biometric shirt's built-in sleep position classification algorithm. With a balanced accuracy of 0.76 and a weighted F1 score of 0.85, we found that the Hexoskin sleep position algorithm was fairly accurate. Our sub analysis, after exclusion of intermediate positions, showed even more promising results (balanced accuracy of 0.80 and weighted F1 score of 0.86). Although excluding intermediate positions improved the overall detection accuracy of the Hexoskin algorithm, it is, however, possible that some of these transitional positions could evolve into a prone position during a seizure. This limitation suggests that intermediate postures should be further studied, to better capture clinically relevant position changes during a seizure.

Most false negatives in detecting prone and supine positions were identified by the Hexoskin algorithm as lateral positions (left and right decubitus). Similarly, most false negatives in detecting lateral decubitus positions were incorrectly identified as supine or prone positions. This issue may be due to the placement of Hexoskin's accelerometer on the right side of the shirt (rather than centrally) and at waist level (rather than at the trunk). Because the device was not centered at chest level, its initial orientation could have varied depending on the patient's waist size. Additionally, the device could have shifted during the night due to the shirt twisting at the waist. Furthermore, there are some sleep positions that require a differentiation between orientation of the hips and the trunk; for instance, the hips might face sideways while the trunk points upward or downward. In such cases, our observation of upper-body position rather than hip orientation could have led to incorrect identifications. This would explain why there is worse detection of supine/prone positions compared to right/left positions. Studies by Ferrer-Lluis et al. (10, 11) showed high accuracy in detecting sleep positions when the accelerometer was centered at chest level, suggesting that some false negatives could have been avoided by placing the device against the sternum (12). The fact that the accelerometer was positioned on the right side of the shirt could also explain why the right-side position was detected more reliably than the left side.

A few other devices have previously been tested for their accuracy in detecting body positions (13). In 2015, Yoon et al. published a study on sleep posture estimation using a triaxial accelerometer in a patch-type device, reporting 99.16% agreement and a Cohen's kappa of 0.98 based on polysomnography (PSG) position sensors in 13 subjects. However, their results were based on non-overlapping 30-s sliding windows during single-night PSG sessions, selecting the most frequent position within each epoch (9). In contrast, our study systematically reviewed 1,249,633 s of sleep on a second-by-second basis, validated against VEEG in an EMU setting. This approach captured (1) night-to-night variability, (2) hospital environment adaptation, (3) greater sleep duration and more frequent position changes, (4) reduced discomfort compared to PSG, which can bias sleep posture, and (5) the absence of imposed sleep position recommendations (as it is often done in PSG). Ferrer-Lluis et al. (11) investigated the feasibility of using smartphone accelerometers strapped to the sternum to monitor sleep positions. Using overnight PSG in six subjects, they reported agreement rates of 100% for supine, 94% for left lateral, 87% for right lateral, and 0% for prone positions. Detection of prone posture was unsatisfactory, likely due to the brief duration of prone positioning (only 30 min analyzed) and the possibility that some PSG-identified prone positions were artifacts. Furthermore, to our knowledge, PSG positions were not video-validated in this study (11). The following year, the same group published a study detailing a new algorithm to detect sleep positions using smartphones. They assessed its accuracy against video-validated PSG in 19 subjects, reporting a high overall system accuracy of 98.2%, but a low sensitivity of 38.9% for prone positions, likely due to limited prone sleep (157.7 min out of 8,155.3 min). As a result, the system was not considered suitable for SUDEP monitoring (10). In 2022, Kukwa et al. (12) evaluated the performance of the Clebre wireless neck-based audio-motion sensor for detecting body positions compared to PSG. The device demonstrated high accuracy, with agreement rates between 96.9% and 98.6% for supine, prone, and lateral positions. However, only four gross positions were analyzed, simplifying the classification problem due to the reduced dimensionality of the output space. Although the study included a relatively large cohort of 89 subjects, it was limited to single-night PSG recordings with a minimum of 6 h per subject. Sleep positions were derived from PSG raw data and only video-validated when discrepancies arose between PSG and Clebre outputs. In 2022, Abdulsadig et al. (13) evaluated two methods for detecting sleep positions using a neck-based accelerometer: a threshold-based model (95% accuracy, 0.89 F1-score) and an Extra-Trees Classifier machine-learning algorithm (99% accuracy, 0.99 F1-score). Of the 18 participants, 13 were used for training and 5 for testing. Subjects were instructed to lie in four main positions (left, right, supine, prone) for at least 30 s each, following verbal cues. These timed positions served as the reference for model evaluation. Additionally, a reference point for acceleration angle calculation was established for each subject during the first supine position. The controlled position changes, initial calibration, and model optimization likely contributed to the near-perfect results. Due to large methodological differences between previously mentioned studies and our work, it is rather difficult to compare their performances.

Our study presents both strengths and limitations. To our knowledge, our study was the first evaluation of a fixed-and-frozen sleep position classification algorithm implemented within a wearable device conducted in an EMU. Our study design allowed for testing of out-of-distribution (OOD) generalization of the performances of the algorithm (independently developed by the manufacturer) (21). While the number of participants was relatively small (10), we had close to 350 h of data over 40 nights to analyze. Because the study was conducted in a hospital setting, the unfamiliar and possibly uncomfortable environment could have increased the prevalence of certain sleeping positions while lessening that of position changes during sleep; moreover, the equipment used for EEG recordings may have limited also positions and movements. However, the purpose of this study was not to determine in what position PWE tend to sleep but to evaluate the performance of the sleep body position algorithm of the Hexoskin shirt. As this study was designed an exploratory validation of the pre-existing algorithm, no formal power analysis was performed. Future studies should confirm these findings in larger patient cohorts in different hospital or residential environments.

Although our study demonstrated that the algorithm can effectively track body position during regular sleep, its performance following a tonic-clonic seizure remains uncertain, as strong convulsions could potentially displace the accelerometer. However, this limitation is mitigated by our prior findings showing that the Hexoskin smart shirt can reliably detect tonic-clonic seizures, thereby enabling rapid intervention regardless of post-ictal body position tracking (14).

Finally, this study presents a preliminary validation of the body position detection algorithm using Hexoskin's shirt. Findings show reliable performance of the algorithm when the device is properly worn. This shows promise in using the biometric shirt to alert patients and caregivers in real-time when a prone sleeping position is detected, enabling timely intervention. The biometric shirt also allows to objectively track sleeping habits, and assess the need for sleep training to influence an ideal sleep position.

## Conclusion

In conclusion, we report that the Hexoskin biometric shirt is fairly reliable in detecting sleep positions in PWE in our EMU. Future steps include evaluating its performance in home settings with slight enhancements such as placing the accelerometer at the center of the upper body.

## Data Availability

The datasets presented in this article are not readily available because sharing of raw data is conditional to approval by our Institution's Research Ethics Board. Requests to access the datasets should be directed to Elie Bou Assi, elie.bou.assi.chum@ssss.gouv.qc.ca.
